# Case Report: Osteomyelitis in a giant panda (*Ailuropoda melanoleuca*)

**DOI:** 10.3389/fvets.2025.1574668

**Published:** 2025-10-09

**Authors:** Chengdong Wang, Haidi Yang, Kai Wu, Linhua Deng, Chengyao Li, Rongping Wei, Caiwu Li, Yan Zhu, Ming Wei, Zhi Huang, Yanxi Cheng, Xuemei Chen, Desheng Li

**Affiliations:** China Conservation and Research Center for the Giant Panda, Key Laboratory of SFGA on the Giant Panda, Chengdu, Sichuan, China

**Keywords:** giant panda (*Ailuropoda melanoleuca*), osteomyelitis, MRI, rifampicin, chitosan

## Abstract

Osteomyelitis is an inflammatory disease of bone tissue induced by microbial infection, and poses a significant health burden in humans and animals. The global annual incidence in humans is estimated ~1–10 cases per 100,000 individuals, with notably higher rates observed in high-risk populations. In domestic cubs and companion animals, trauma-related osteomyelitis can occur at an incidence rate ranging from 0.1 to 5%. Although osteomyelitis is well-documented in both human and veterinary medicine, with diverse and complex diagnostic and therapeutic approaches available for human cases, it has not been previously reported in the giant panda (*Ailuropoda melanoleuca*). This report presents a case of osteomyelitis in an elderly giant panda exhibiting lameness in the left hind limb. Diagnosis was confirmed trough laboratory testing, computed tomography (CT), and magnetic resonance imaging (MRI). The animal underwent a 2-month course of treatment with rifampicin (Guangzhou Baiyunshan Pharmaceutical Group Co., LTD) and medical-grade chitosan (Qingdao Jintieshan Biotechnology Co., LTD), which resulted in significant clinical improvement. This retrospective case analysis provides valuable insights into the clinical diagnosis and management of osteomyelitis in giant pandas and contributes to the foundational knowledge necessary for the prevention and treatment of this condition in captive populations.

## 1 Introduction

The giant panda (*Ailuropoda melanoleuca*), as a flagship species for global biodiversity conservation, requires meticulous health management, which is essential for the survival of this endangered species.

Osteomyelitis, a prevalent disease affecting both humans and vertebrates, poses significant threats to their health (1). It refers to an inflammation of the bone tissue, usually caused by bacterial infections, though fungal or viral pathogens can also be responsible (2, 3). Epidemiology shows humans like infants, the elderly and those with compromised immune systems are more susceptible (4). This disease is not exclusive to humans. In other animals, for example, dogs often suffer from osteomyelitis due to open fractures, bites from other animals, or spread of infections from other parts of the body (5, 6). Cats can also be affected, with juvenile cats being more prone to acute forms (7), while older cats may develop chronic osteomyelitis. In livestock such as cattle and sheep, osteomyelitis can occur as a result of injuries during grazing or fighting, and it may lead to significant economic losses in the farming industry (8, 9). In the context of wildlife conservation, understanding osteomyelitis is crucial for the health management of endangered species like the giant panda. The symptoms and signs of osteomyelitis patients typically include swelling, warmth, redness at the infected site, pain, and mobility difficulties (10). However, a significant diagnostic challenge lies in the fact that sometimes osteomyelitis may present with no obvious signs or symptoms, or the symptoms are easily confused with other diseases (11). As for giant pandas, due to the unique characteristics of the species-their thick fur can mask localized swelling or redness and their pain can be masked and they may hide signs of pain–accurately identifying these symptoms can be even more difficult.

Laboratory tests play a role in diagnosing osteomyelitis. The majority of osteomyelitis cases are bacterial in origin, and laboratory findings often reveal elevated erythrocyte sedimentation rate (ESR) and C-reactive protein (CRP) levels (12). Relying solely on blood markers may lead to misdiagnosis as they can be elevated in various inflammatory conditions. Furthermore, conventional imaging modalities such as radiography (X-ray), computed tomography (CT), and magnetic resonance imaging (MRI), are also used in clinical assessment. Nevertheless, accurate diagnose across different stages of osteomyelitis remains a clinical challenge (13). Bone destruction usually needs to reach 30%−50% to be detected on X-rays, so X-rays may not detect early-stage osteomyelitis, because soft tissue swelling and reduced mineral opacity may be subtle and can be overlooked during this period (14, 15). Computed tomography (CT) is an advanced imaging technique based on X-ray technology that provides high-resolution cross-sectional images of internal anatomical structures. A key advantage of CT is its rapid imaging acquisition enabling comprehensive scans to be completed in a short time, which is an essential feature for prompt diagnosis in a critical care setting (16, 17). In veterinary medicine, this rapidity is particularly valuable given the challenges of anesthetizing non-human species—wild animals often tolerate anesthesia poorly (18), and CT's short scan times reduce risk of anesthesia related complications. While CT provides excellent visualization of subchondral bone and cortical bone, its ability to assess tendinous and ligamentous soft tissues is limited, particularly without contrast enhancement (19, 20). Equine studies have demonstrated that contrast-enhanced CT improves the evaluation of tendons and ligaments. In addition, CT can depict early inflammatory changes, such as loss of fat planes and soft tissue swelling. Therefore, CT should be considered to complementary to other imaging modalities rather than a stand-alone technique for soft tissue assessment. In the early stages of osteomyelitis, CT may fail to demonstrate subtle findings such as early bone marrow edema or the slightest periosteal reaction (21). MRI, which uses magnetic fields and radio frequency pulses, provides excellent soft tissue contrast (22). When used in combination, MRI and CT enhance diagnostic accuracy by providing complementary information: CT excels in delineating bony architecture, whereas MRI is highly sensitive to marrow edema and soft tissue inflammation. Both modalities are widely applied in the clinical diagnosis of osteomyelitis and are well-documented in the literature (23–25).

This report for the first time details the diagnosis and symptomatic treatment of osteomyelitis in an elderly giant panda with lameness through hematology, biochemistry, computed tomography (CT), and MRI.

## 2 Case presentation

**Figure 1:**
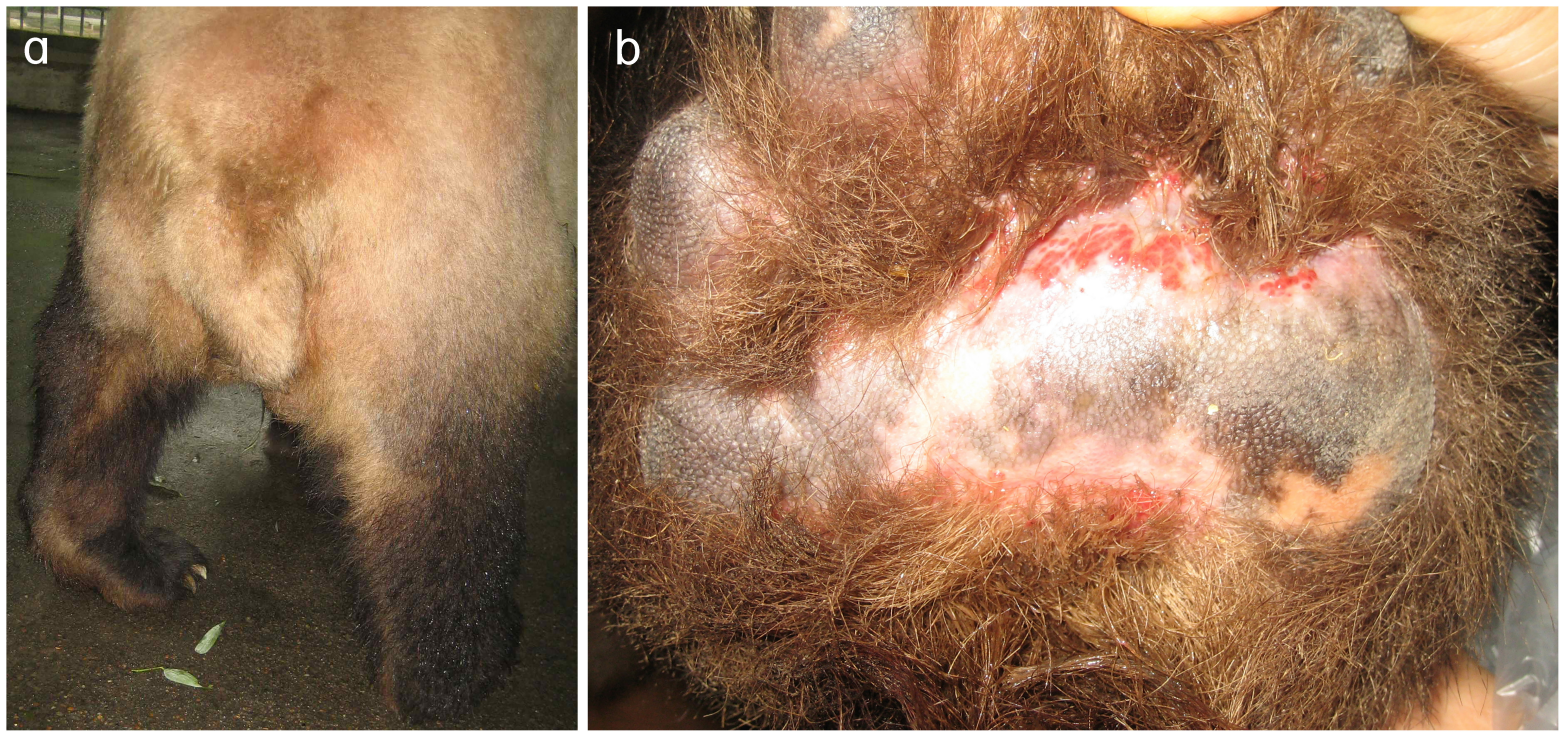
Clinical manifestations of the left hind limb in the affected giant panda. **(a)** Left hind limb of the giant panda. During ambulation, the plantar surface fails to make contact with the ground, resulting in severe locomotor dysfunction. **(b)** Abraded left hind paw pad of the giant panda. Prolonged abnormal weight-bearing avoidance led to redness and swelling.

A male giant panda, ~26 years old and weighing around 120 kg, was previously healthy with no significant medical history. This animal developed acute lameness, which significantly restricted mobility. Subsequently, within and a few days, the giant panda was unable to completely bear weight on the left hind limb (Figure 1a). In response to these concerning symptoms, the veterinary team decided to initiate a comprehensive clinical examination.

**Figure 2:**
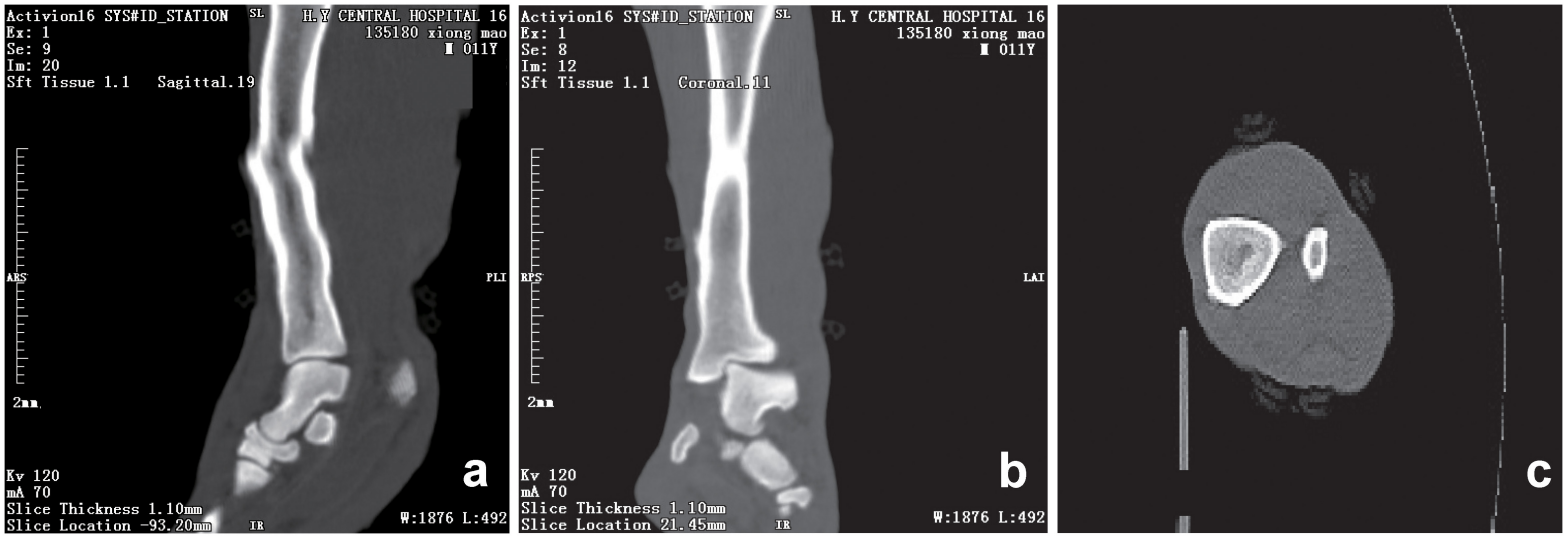
CT scans of the hind limb of the giant panda. Sagittal **(a)**, coronal **(b)** and transverse **(c)** computed tomography (CT) scan of the left hind limb showing smooth, well-defined articular margins. The tarsal bones are correctly aligned, and corticomedullary definition is normal. The distal tibial articular surface shows a normal inclination angle. No fractures are observed. The osseous structures of the included joints are intact with no adjacent significant soft tissue swelling. A slight step artifact is present in the mid and distal diaphysis of the tibia. Scan parameters: tube voltage = 120 kV; tube current = 70 mA; slice thickness = 1.10 mm; window width (WW) = 1,876 HU; window level (WL) = 492 HU; reconstructed using a soft tissue 1.1 sequence.

**Figure 3:**
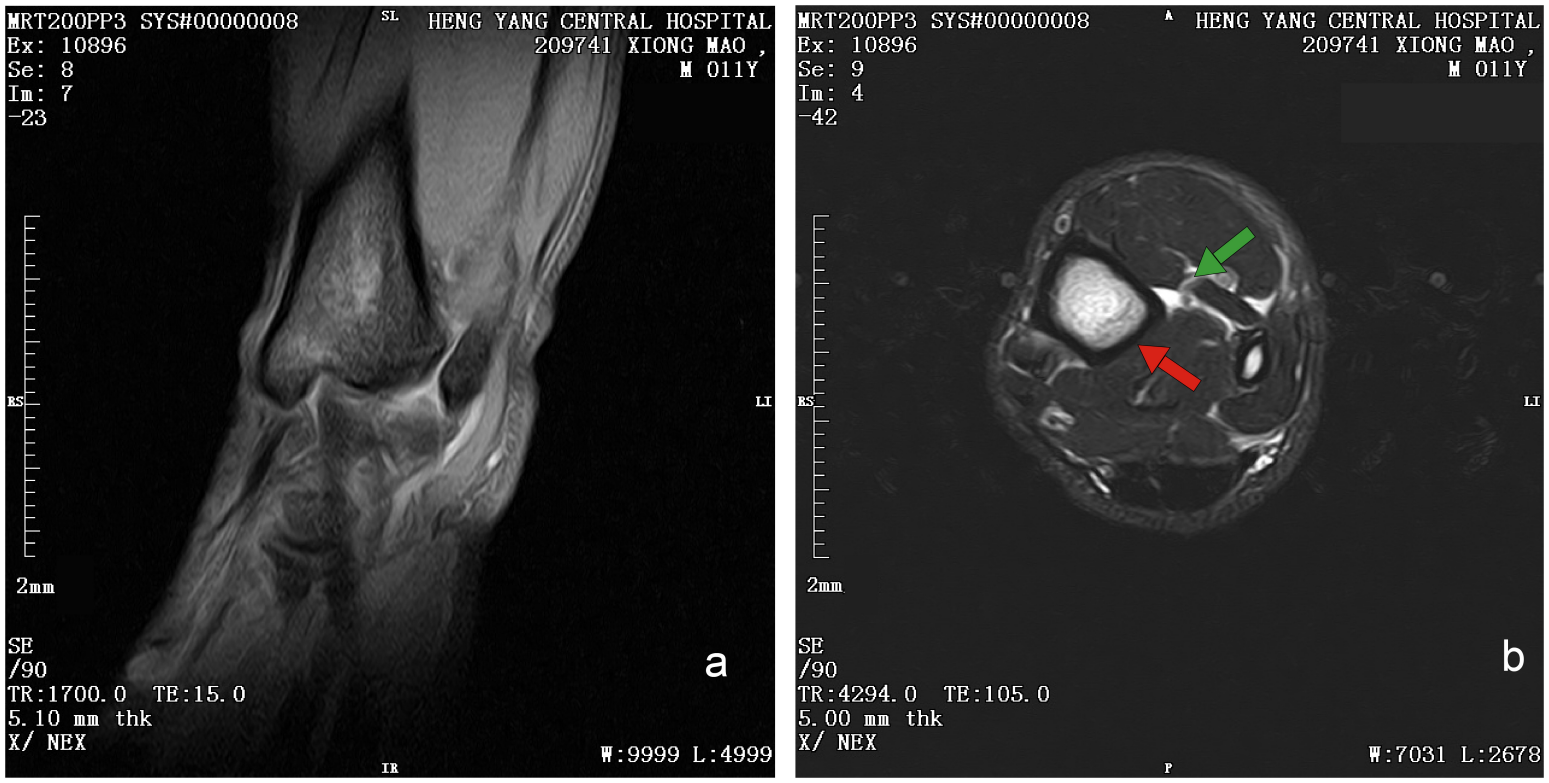
Magnetic resonance image of left hind limb of giant panda. **(a)** T1W sequence of sagittal plane. Sequence Parameters: repeat time (TR) of 1,700 ms, echo time (TE) 15 ms, slice thickness of 3.8 mm, window width (W) of 6,438 HU, and window level (L) of 3,192 HU. Bony structures (distal tibia, talus, calcaneus) show clear margins with homogeneous bone marrow signal (isointense to muscle on T1W). Articular cartilage appears smooth with isointense signal; No definite bony cortex disruption or abnormal signal involvement of bones. **(b)** T2W sequence of transverse plane. Sequence Parameters: repeat time (TR) of 4,294 ms, echo time (TE) 105 ms, slice thickness of 5 mm, window width (W) of 7,031 HU, and window level (L) of 2,678 HU. Patchy hyperintensities (long T2 signal, suggestive of edema/inflammation) are noted in soft tissues around the distal tibia (intermuscular space); Focal hyperintensities are observed in soft tissues posterior to the calcaneus (around the Achilles tendon), with clear demarcation from the tendon.

Initial clinical evaluation involved observation and palpation of the limbs, followed by blood collection for routine hematological and biochemical testing. A non-anesthetic examination revealed mild atrophy of the left hind limb, with no pain response on palpation. There was mild alopecia on the medial aspect of the left hind limb's thigh and increased skin temperature, without detected swelling, trauma, or joint deformity. Additionally, claw abrasion was noted on the right paw, accompanied by erythema and swelling of the right paw pad (Figure 1b). No other abnormalities were detected during this initial assessment. Blood samples were collected concurrently for routine blood and biochemical tests, which subsequently revealed low lymphocyte and platelet counts, and the levels of total cholesterol (TC) and triglycerides (TG) were elevated. However, no significant increase in C-reactive protein (CRP) was observed (since there was no reference range for CRP in giant pandas, human levels were used here; Table 1). Throughout this examination phase, the panda's diet and weight remained stable, consistent with its baseline status. 3 days later, under general anesthesia, further examination was performed. The panda's body temperature was normal, and no external injuries, or draining fistulas were observed. Subsequently, advanced imaging modalities were employed. Non-enhanced scanning of both hind limbs was performed using a Toshiba Activion 16 multislice CT scanner (TOSHIBA, Tokyo, Japan). The panda was placed in a dorsal recumbency. The scanning protocol included both hindlimbs, extending from the femur to the phalanges. A helical scan mode was used with a gantry rotation time of 0.5 s per rotation and a detector configuration of 16 rows × 1.0 mm. The beam collimation width was matched to the detector configuration (16 × 1.0 mm), with an original slice thickness of 1.0 mm. Tube voltage was set at 120 kV and tube current at 70 mA. The pitch was 1.375:1. The scan field of view (SFOV) was adjusted according to the number of hind limbs: 450 mm for bilateral and 400 mm for unilateral examinations. The *Z*-axis scanning range was ~600 mm, and the original slice thickness was fixed at 1.0 mm. Image reconstruction parameters were tailored to diagnostic needs. For bone evaluation, images were reconstructed with a slice thickness of 5.0 mm. For soft tissue evaluation, images were reconstructed with a slice thickness of 1.1 mm and a reconstruction interval of 0.69 mm. A high-resolution bone algorithm (Bone 5.0) was applied for bone reconstruction, and a standard soft tissue algorithm (Soft Tissue 1.1) was applied for soft tissue reconstruction. The reconstruction matrix was uniformly 512 × 512, with a pixel size of 0.8 mm. The display field of view (DFOV) was set to 350 mm. For the bone window, window width and level were 2,000 HU and 500 HU, respectively; for the soft tissue window, window width and level were 1,876 HU was 492 HU, respectively. CT scans revealed no evidence of fractures, joint space narrowing or widening, osteophyte formation, or other bony abnormalities in either hind limb (Figure 2). With the permission of the government, these CT scans were read jointly by radiologists and veterinarians, who confirmed that there were no osseous abnormalities in the two hind limbs. Following the CT examination, a Toshiba Vantage Titan 1.5T MRI (TOSHIBA, Tokyo, Japan) was used to perform MRI examinations. Image acquisition is carried out with a slice thickness of 3–5 mm and an interval of 1–2 mm. First, conventional SE sequences were used for image acquisition (T1W: TR 1,700 ms, TE 15 ms); T2W: TR 4,294 ms, TE 105 ms). T1W sagittal images of the tarsus show that the bony structures, including the distal tibia, talus, and calcaneus, have distinct and well-defined margins. The bone marrow signal within these bones is homogeneous, with no apparent abnormal hypointense or hyperintense areas detected. The articular cartilage of the tarsus maintains a smooth surface, and its signal intensity on T1W imaging is consistent with that of normal articular cartilage, appearing isointense relative to the adjacent muscle tissue in most regions. The tarsus ligaments, such as the dorsal talofibular ligament and calcaneofibular ligament (visualized in appropriate sections), exhibit normal low–signal characteristics on T1W sequences, without evidence of discontinuity, thickening, or abnormal signal intensity. The surrounding soft tissues, including the tendons (e.g., the Achilles tendon, tibialis cranial) and the joint capsule, display a normal layered structure, with no significant swelling, thickening, or abnormal signal intensity changes. The T2W transverse image shows that patchy high signals are found in the soft tissues around the distal tibia and the plantar aspect of the tarsus calcaneus, with indistinct edges. The signal intensity is higher than that of the adjacent normal muscle tissue. The high intensity around the distal tibia is mainly located in the interosseous space or interosseous muscle on the inner side of the distal tibia. The high signal plantar calcaneus is mainly distributed around the subcutaneous soft tissue on the inner, outer and plantar sides of the Achilles tendon (Figure 3). At the same time, there are also some high signal intensities in soft tissues of the plantar aspect of tarsus. Based on MRI findings, a diagnosis of osteomyelitis in the left hindlimb was made.

**Table 1:** Routine blood and biochemical test report of giant panda.

**Terms**	**Code name**	**Results**	**Reference range**	**Evaluation**
White blood cell count	WBC	6.0	7.88 ± 1.34 × 10^9^/L (39)	–
Lymphocyte count	LY^#^	1.8	1.08 ± 0.34 × 10^9^/L (40)	↑
Monocyte count	MONO^#^	0	1.43 ± 0.85 × 10^9^/L (41)	↓
Neutrophil count	NEUT^#^	4.2	4.85 ± 1.17 × 10^9^/L (39)	–
Lymphocyte ratio	LY%	30	27.02 ± 3.81% (39)	–
Monocyte ratio	MONO%	0	4.59 ± 1.15% (39)	↓
Neutrophil ratio	NEUT%	70	68.80 ± 3.37% (39)	–
Hemoglobin	HGB	104	119.98 ± 6.36 g/L (39)	↓
Red blood cell	RBC	6.39	6.12 ± 0.31/L (39)	–
Hematocrit	HCT	32	33.32 ± 1.76% (39)	–
Mean corpuscular volume	MCV	50.1	54.78 ± 1.37 Fl (39)	↓
Mean corpuscular hemoglobin	MCH	20.9	19.62 ± 0.41 PG (39)	–
Mean corpuscular hemoglobin concentration	MCHC	418	358.48 ± 4.63 g/L (39)	↑
Coefficient variation of red blood cell volume distribution width	RDW-CV	14.8	16.33 ± 0.90% (39)	–
Standard deviation of red blood cell volume distribution width	RDW-SD	25.9	33.20 ± 1.53% (39)	↓
Platelet count/blood platelet count	PLT	270	525.77 ± 96.45 × 10^9^/L (39)	↓
Mean platelet volume	MPV	5.1	6.05 ± 1.04 Fl (40)	–
Platelet distribution width	PDW	15.3	9.93 ± 4.64 Fl (42)	↑
Thrombocytocrit	PCT	0.137	0.31 ± 0.05% (39)	↓
Potassium	Ka	5.6	4.83 ± 0.43 mmol/L (39)	–
Sodium	Na	125.3	129.09 ± 1.36 mmol/L (39)	–
Chlorine	Cl	97.7	97.44 ± 2.68 mmol/L (39)	–
Calcium	Ca	2.1	2.07 ± 0.11 mmol/L (39)	–
Phosphorus	P	0.12	1.32 ± 0.12 mmol/L (39)	↓
Magnesium	Mg	0.79	0.99 ± 0.11 mmol/L (39)	–
Iron	Fe	20.3	30.80 ± 4.24 mmol/L (39)	↓
Carbon dioxide combining power	CO_2_-CP	21.0	22.32 ± 1.78 mmol/L (39)	–
Blood urea nitrogen	BUN	4.1	7.08 ± 1.15 mmol/L (39)	↓
Creatinine	Cr	85.3	101.90 ± 26.34 mmol/L (39)	–
Blood urea nitrogen/creatinine	BUN/Cr	0.05	0.09 ± 0.04 (39)	–
Uric acid	UA	20.3	56.68 ± 14.62 mmol/L (39)	↓
Glucose	GLU	3.4	3.22 ± 0.40 mmol/L (39)	–
Osmotic pressure	OP	265.2	260–320 OSM (homo)	–
Triglyceride	TG	2.2	1.33 ± 0.31 mmol/L (39)	↑
Total cholesterol	TC	5.7	4.88 ± 1.15 mmol/L (39)	↑
High density lipoprotein	HDL	4.2	2.96 ± 0.47 mmol/L (40)	↑
Low-density lipoprotein	LDL	0.46	3.41 ± 1.46 mmol/L (40)	↑
Apolipoprotein A	ApoA	0.183	0.71 ± 0.18 g/L (40)	↓
Apolipoprotein B	ApoB	0.005	0.04 ± 0.03 g/L (40)	↓
Total protein	TP	74.9	65.83 ± 3.07 g/L (39)	↑
Albumin	ALB	30.3	34.31 ± 2.38 g/L (39)	↓
Globulin	GLB	44.6	31.52 ± 3.79 g/L (39)	↑
Albumin/globulin	ALB/GLB	0.68	1.12 ± 0.17 (39)	↓
Alanine transaminase	ALT	42.0	81.76 ± 19.84 U/L (40)	↓
Aspartate aminotransferase	AST	67.3	71.93 ± 13.34 U/L (40)	–
Aspartate aminotransferase/alanine transaminase	AST/ALT	1.6	0.91 ± 0.18 (40)	↑
Total bilirubin	T-BIL	3.5	1.06 ± 0.29 μmol/L (39)	↑
Direct bilirubin	D-BIL	1.2	0.51 ± 0.18 μmol/L (39)	↑
Indirect bilirubin	I-BIL	2.3	0.55 ± 0.22 μmol/L (39)	↑
Total bile acids	TBA	72.3	75.35 ± 10.18 μmol/L (42)	–
Alpha-hydroxybutyrate dehydrogenase	α-HBDH	833.6	548.30 ± 297.50 U/L (39)	–
Lactate dehydrogenase	LDH	951.8	632.59 ± 329.83 U/L (39)	–
Creatine phosphokinase	CPK	175.8	194.96 ± 45.77 U/L (39)	–
Creatine kinase isoenzyme	CPKIso	290.4	260.83 ± 67.56 U/L (39)	–
Myoglobin	Mb	23.0	0–100 μg/L (homo)	–
Cholinesterase	CHE	694.8	766.25 ± 232.49 (42)	–
Alkaline phosphatase	ALP	116.1	117.07 ± 38.60 U/L (40)	–
γ-glutamyltranspeptidase	GGT	0.2	10.54 ± 6.26 U/L (40)	↓
α-L-fucosidase	AFU	2.6	4.64 ± 1.08 IU/L (40)	↓
Lipase	Lip	14.2	0–60 IU/L (homo)	
Amylase	AMY	1,301.5	1,138.26 ± 386.59 U/L (39)	–
Cystatin c	CYS-c	0.21	0.54 ± 0.18 mg/L (41)	↓
Hypersensitive C-reactive protein	Hs-CRP	0.37	0–3mg/L (homo)	–
Adenosine deaminase	ADA	8.5	5.47 ± 1.38 U/L (40)	↑
Beta-hydroxybutyric acid	β-HB	0.06	0.03–0.3 μmol/L (homo)	–
5′nucleotidase	5′-NT	0.6	2.24 ± 1.06 U/L (40)	↓
Ischaemic modified albumin	IMA	61.0	> 64.7 U/ml (homo)	↓

The routine blood test of this giant panda was carried out on Mindray BC5500 (Mindray Medical International Co., LTD, Shenzhen, China) and the blood biochemistry test of this giant panda was carried out on Mindray BS-600M (Mindray Medical International Co., LTD, Shenzhen, China). The reference range of each indicator is detailed in the reference. ^#^=count.

On the second day after the diagnosis, treatment was initiated. The giant panda was administered three cefixime capsules (100 mg per capsule, Guangzhou Baiyunshan Pharmaceutical Group Co., LTD, 2.5 mg/kg) twice daily. After 1 week of continuous administration, noticeable improvement was observed in the left hind limb. The pandas could exhibit normal plantigrade stance with the entire plantar surface of the hindlimbs touching the ground, and their overall activity level also increased. This situation persisted for 4 weeks without any further significant improvement. In response to this, the veterinarian adjusted the treatment plan, continuing the administration of three cefixime capsules (p.o., 2.5 mg/kg) twice daily and adding daily hind limb massages for 1 week. 3 days after this adjustment, the panda's condition improved, with a gradual increase in activity. Although the tiptoed gait persisted, the panda was able to support its body weight with both hind limbs. Given the instability of the condition, the medication was changed to rifampicin and chitosan 1 week later. Rifampicin (150 mg per capsule, Guangzhou Baiyunshan Pharmaceutical Group Co., LTD) and chitosan (200 mg per capsule, Qingdao Jintieshan Biotechnology Co., LTD) were administered orally, with five capsules of each taken on an empty stomach in the morning, and an additional five chitosan capsules (200 mg per capsule) given in the afternoon.

After 2 months of continuous treatment with the adjusted medication regimen, the symptoms were substantially relieved, and the medication was discontinued. 1-week post-discontinuation, no lameness and swelling were observed in the hind limbs. Moreover, the panda's overall demeanor, spirit, and appetite remained consistently good, and its fasting weight was maintained at 119 kg, indicating a successful resolution of the osteomyelitis episode and a return to a stable health status. During the subsequent long-term feeding and management until its death, we did not find any abnormalities in its two hind limbs.

## 3 Discussion

Osteomyelitis, characterized by inflammation of the periosteum, sclerotin, and bone marrow, and it is caused by purulent bacteria (26). Based on the disease progression, it can be categorized into acute and chronic forms. Acute osteomyelitis progresses rapidly, often accompanied hyperpyrexia and toxemia. Typical clinical signs and manifestations of infection include erythema, swelling, warmth, and pain. Blood examinations usually reveal elevated leukocyte and neutrophil counts. Notably, due to the evolving virulence of pathogenic bacteria and enhanced host resistance, subacute or chronic osteomyelitis frequently occurs, presenting with insidious symptoms such as joint pain in the affected bone or adjacent joints. This poses significant challenges to clinical diagnosis (1, 13).

In general, acute osteomyelitis is associated with elevated leukocytes, neutrophils, and C-reactive protein (CRP) (27). However, in this case, laboratory clinical examination showed no increase in leukocytes, neutrophils, or CRP, strongly suggesting that the condition was not acute. An increase in TC and TG indicates hyperlipidemia and requires dietary adjustments. However, these indicators are likely not associated with the onset of osteomyelitis.

CT provides high density resolution, enabling clear detection of bone damage, particularly cortical bone destruction Early subperiosteal abscess formation can be identified by quantitative analysis of lesion attenuation values (28). However, conventional CT, as used in the present study, has limited sensitivity for detecting early marrow involvement in osteomyelitis. In its early stages, osteomyelitis may lack characteristic findings such as lysis, soft tissues changes, or clear inflammatory infiltration, making accurate localization and assessment of lesion extent challenging (29). Notably, dual-energy CT—an advanced modality capable of detecting bone edema—was not available for this study. Although quantitative analysis of CT attenuation values may improve detection of early lesions, this was not performed in the present cases. Consistent with the limitations of conventional CT in identifying subtle early changes, CT findings were normal, prompting additional MRI examination to clarify the condition. MRI, provides higher soft tissue resolution and contrast, excelling in detecting early marrow changes, making it a preferred for early diagnosis of osteomyelitis (30). The MRI results of the giant panda's lower limb showed isointense signals on T1WI, indicating normal bone morphology, which was consistent with the CT findings. However, the high signal on T2WI indicated the presence of a lesions. Given that this giant panda had no recent history of trauma, and its MRI scans revealed no trauma-specific imaging features—such as fracture lines, acute bone marrow contusions with diffuse hemorrhagic signals, or signs of soft tissue laceration—these findings effectively ruled out traumatic abnormalities in bone marrow signals. Similarly, no masses were noted on imaging, suggesting the absence of neoplastic lesions. Thus, the combined use of CT and MRI in this giant panda case highlights their complementary roles in diagnosing bone and soft tissue abnormalities. While CT effectively rules out gross bony lesions, MRI's superior sensitivity to early marrow changes and soft tissue pathology enables the detection of subtle lesions that may indicate early-stage osteomyelitis.

Upon diagnosing osteomyelitis, veterinarians opted for conservative oral antibiotic treatment to prevent disease progression in this geriatric giant panda, as surgical and intravenous therapies posed high risks (e.g., anesthesia complications, management challenges).

Rifampicin, a broad-spectrum antibiotic, inhibits bacterial RNA polymerase, acting against pathogens such as *Staphylococcus aureus* and *Mycobacterium tuberculosis* (31). Chitosan, a natural polysaccharide, exhibits anti-inflammatory, tissue repair-promoting, immunomodulatory, and moderate antibacterial properties (32, 33). The combined application of rifampicin and chitosan has been reported to be effective in the treatment of osteomyelitis, especially in cases of bacterial L- form infections or hypertrophic osteoarthropathy resulting from inappropriate antibiotic selection, with a low incidence of adverse reaction (34). Moreover, it has shown unique therapeutic effects in subacute and chronic osteomyelitis of human (35). The pharmacological mechanism of their combined is based on a triple approach of “antibacterial–anti-inflammatory–repair-promoting,” which enhances bacterial clearance, reduce inflammatory damage, and promotes bone tissue regeneration. In veterinary medicine, the use of this combination remains primarily experimental, as there is currently no established standard practice for its application in wildlife. Supporting evidence in veterinary species is limited but includes extrapolation from human clinical data and preliminary studies in domestic mammals (e.g., rabbits, horses) demonstrating comparable antibacterial and tissue-repair effects (36–38). For this case, rifampicin and chitosan were administered separately (not as compound preparation). The administration regimen for giant pandas was based on the animal's weight (~120 kg) and administered by referring to the therapeutic dose in humans or in human patients. In addition, based on the clinical experience of the veterinary team in treating captive giant pandas with antibiotics, the dosage was fine-tuned to balance efficacy and safety.

The combination therapy yielded positive outcomes, with the panda's lameness resolving and no recurrence observed after discontinuation. However, this case was constrained by significant limitations: bacterial culture was not performed due to challenges in obtaining adequate samples (e.g., avoiding iatrogenic injury to the giant panda during sample collection from deep bone lesions), leaving the causative pathogen unidentified. This hindered precise antibiotic selection, manifested as the initial ineffectiveness of antibiotics and reduced the study's scientific rigor.

To address these limitations in future cases, targeted improvements in diagnostic and therapeutic strategies are of paramount importance. For diagnostic advancements, priority should be given to optimizing sample collection protocols: minimally invasive techniques (e.g., ultrasound-guided needle aspiration of abscesses or bone marrow biopsies) should be employed to obtain lesion-derived samples while minimizing stress to the animal. Furthermore, integrating advanced molecular diagnostic tools—such as 16S rRNA gene sequencing or metagenomic analysis—can facilitate the identification of fastidious pathogens that are difficult to culture. Simultaneous implementation of aerobic and anaerobic bacterial cultures, combined with antimicrobial susceptibility testing (AST), will further enable precise pathogen identification and guide evidence-based antibiotic selection, thereby reducing reliance on empirical therapy. In terms of therapeutic refinement, future protocols should emphasize individualized dosing strategies tailored to the unique physiological characteristics of giant pandas. This includes leveraging pharmacokinetic studies in captive giant pandas or phylogenetically related species (e.g., bears) to establish species-specific drug metabolism parameters, ensuring optimal dosing of rifampicin, chitosan, or alternative agents. Collectively, these measures will address the current gaps in pathogen identification and therapeutic precision, strengthening the scientific basis for the management of osteomyelitis in giant pandas and other species.

Although this research report has limitations, it represents the first reported case of osteomyelitis diagnosis and treatment in giant pandas. It is hoped that this report can serve as a valuable reference for future clinical diagnosis, treatment, breeding, and management of giant pandas.

## Data Availability

The original contributions presented in the study are included in the article/supplementary material, further inquiries can be directed to the corresponding author.
